# Neoplastic Transformation of Human Mesenchymal Stromal Cells Mediated via *LIN28B*

**DOI:** 10.1038/s41598-019-44536-1

**Published:** 2019-05-30

**Authors:** Radhakrishnan Vishnubalaji, Ramesh Elango, Mashael Al-Toub, Muthurangan Manikandan, Ammar Al-Rikabi, Linda Harkness, Nicholas Ditzel, Muhammad Atteya, Rimi Hamam, Musaad Alfayez, Abdullah Aldahmash, Moustapha Kassem, Nehad M. Alajez

**Affiliations:** 1 Cancer Research Center, Qatar Biomedical Research Institute (QBRI), Hamad Bin Khalifa University (HBKU), Qatar Foundation (QF), PO Box 34110, Doha, Qatar; 2 College of Applied Medical Sciences, King Saud University, Riyadh, 11461 Saudi Arabia; 3 Stem Cell Unit, Department of Anatomy, College of Medicine, King Saud University, Riyadh, 11461 Saudi Arabia; 4 Department of Pathology, King Saud University Medical City, Riyadh, Saudi Arabia; 5 Australian Institute for Bioengineering and Nanotechnology, The University of Queensland, Queensland, Australia; 6 Molecular Endocrinology Unit (KMEB), Department of Endocrinology, University Hospital of Odense and University of Southern Denmark, Odense, Denmark; 7 Histology Department, Faculty of Medicine, Cairo University, Cairo, Egypt; 8 Departement of Medicine, University of Montreal, Montreal, Canada; 9 Prince Naif Health Research Center, King Saud University, Riyadh, 11461 Saudi Arabia; 10 Department of Cellular and Molecular Medicine, Danish Stem Cell Center (DanStem), University of Copenhagen, 2200 Copenhagen, Denmark

**Keywords:** Oncogenes, Cell growth

## Abstract

Bone marrow stromal (Mesenchymal) stem cells (MSCs) are multipotent bone cells capable of differentiating into mesoderm-type cells, such as osteoblasts and adipocytes. Existing evidence suggests that transformation of MSCs gives rise to sarcoma. In order to identify the molecular mechanism leading to spontaneous transformation of human bone marrow MSCs (hBMSCs), we performed comprehensive microRNA (miRNA) and mRNA profiling in the transformed hBMSC-Tum line compared to the parental clone. As a result, we identified multiple dysregulated molecular networks associated with the hBMSC transformed phenotype. *LIN28B* was upregulated 177.0-fold in hBMSC-Tum, which was associated with marked reduction in *LET-7* expression and upregulated expression of its target *HMGA2*. Targeted depletion of *LIN28B* or exogenous expression of *LET-7b* suppressed hBMSC-Tum proliferation, colony formation, and migration. On the other hand, forced expression of *LIN28B* promoted malignant transformation of parental hBMSC cells as shown by enhanced *in vitro* colony formation, doxorubicin resistance, and *in vivo* tumor formation in immunocompromised mice. Analysis of *LIN28B* and *HMGA2* expression levels in cohorts from The Cancer Genome Atlas sarcoma dataset revealed a strong inverse-relationship between elevated expression and overall survival (OS) in 260 patients (p = 0.005) and disease-free survival (DFS) in 231 patients (p = 0.02), suggesting LIN28B and HMGA2 are important regulators of sarcoma biology. Our results highlight an important role for the LIN28B/LET-7 axis in human sarcoma pathogenesis and suggest that the therapeutic targeting of LIN28B may be relevant for patients with sarcoma.

## Introduction

Sarcomas are malignant tumors that arise from transformed cells of stromal or mesenchymal origin. Despite their rarity, sarcomas represent a clinical challenge requiring a better understanding of their pathogenesis, as well as a need for the identification of novel markers to be employed in targeted therapy^1^.

A number of recent studies have demonstrated that stromal human mesenchymal stem cells (hMSCs) represent the cells-of-origin of sarcomas. We have previously reported transformed phenotype of immortalized primary bone marrow-derived human stromal (mesenchymal) cells upon continuous passaging in culture^2^. Results from other studies are consistent with these initial findings that hMSCs were serially transformed by a retrovirus containing human telomerase reverse transcriptase, simian virus 40 large T antigen (SV40 TAg), and lentivirus containing oncogenic H-Ras, leading to the formation of sarcoma-like tumors *in vivo*^3^.

The transforming property of MSCs and their role as sarcoma-initiating cells suggest their use as a suitable model to investigate sarcoma pathogenesis^4^, and to identify mechanism(s) underlying malignant transformation. Herein, we performed global mRNA and microRNA (miRNA) expression profiling in non-transformed parental hBMSC and transformed hBMSC-Tum cells. Our data revealed distinct miRNA and mRNA expression profiles associated with malignant transformation, highlighted a pivotal role for the LIN28B/LET-7 axis in the transformation process of hBMSC, and indicated that elevated expression of LIN28B and HMGA2 may be predictive of poor clinical outcome in sarcoma patients.

## Results

### Multiple altered genetic pathways were associated with the transformed phenotype of hBMSC-Tum cells

**Figure 1:**
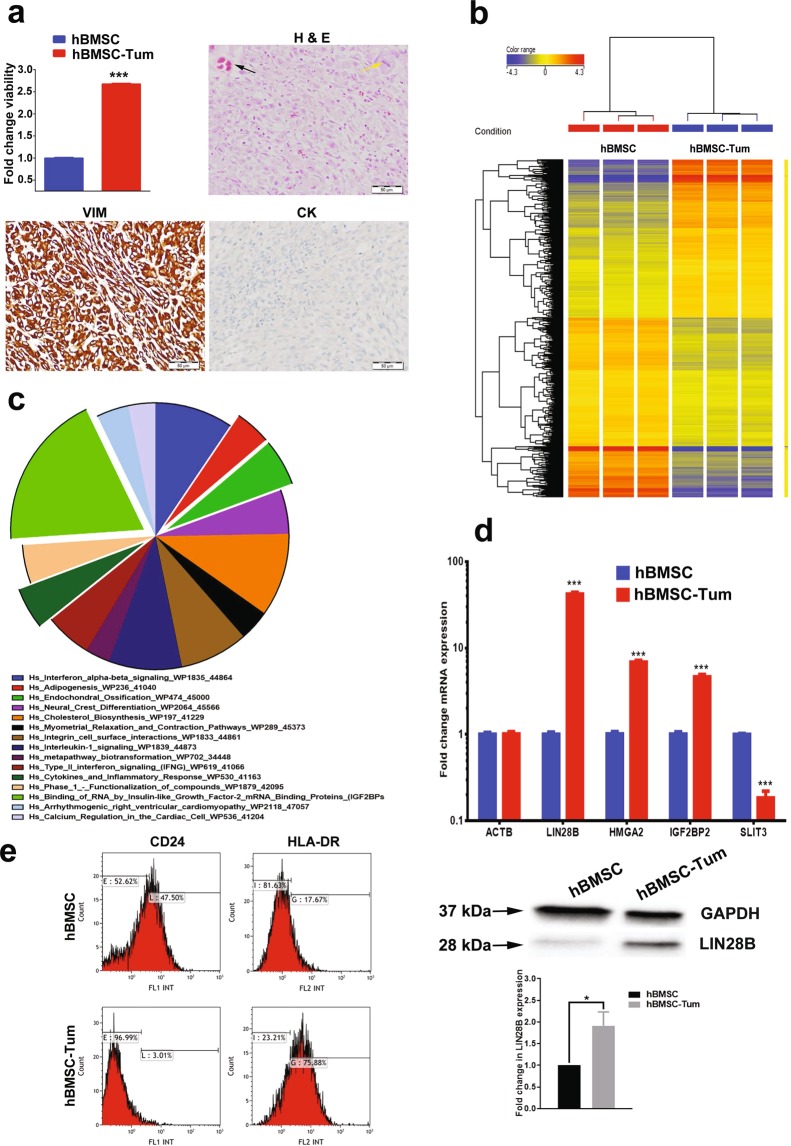
The tumorigenic cell line (hBMSC-Tum) exhibited changes in multiple genetic pathways. The tumorigenic stromal human mesenchymal cell line (hBMSC-Tum) was compared to the non-tumorigenic hBMSC cell line *in vitro and in vivo*. (**a**) Cell viability determined using an alamarBlue assay (upper left panel). Data are presented as the mean fold change ± S.E., n = 48 wells per condition from two independent experiments. ***P < 0.0005. Representative hematoxylin and eosin (H & E) staining for histological examination (upper right panel) showing a sarcoma-like phenotype for the hBMSC-Tum xenograft, the presence of multiple mitotic cells (yellow arrow), and vascularization (black arrow). The tumors were positive for vimentin (lower left) and were negative for cytokeratin (lower right). (**b**) Hierarchical clustering of hBMSC-Tum cells compared to parental hBMSC cells based on differentially expressed mRNA levels as determined by microarray analysis. Each column represents one technical replica and each row represents an mRNA transcript. The expression level of each gene in a single sample is depicted according to the color scale. (**c**) Pie chart illustrating the distribution of the top-15 genetic pathways based on differentially expressed genes in the hBMSC-Tum cells compared to that in the parental hBMSC cells. The size of the chart segment corresponds to the fold-enrichment. (**d**) The expression levels of genes selected from the microarray data validated using qRT-PCR (upper panel). Data are presented as the mean ± S.E., n = 6 technical replicas. ***P < 0.0005. Representative immunoblotting results showing the upregulation in the expression of the LIN28B protein in hBMSC-Tum cells compared to its expression levels in the control cells (lower panel). Quantification of relative protein expression is shown form 3 technical replicas. (**e**) Cell surface antigen expression on hBMSC and hBMSC-Tum cells as shown by FACS analysis.

Few studies have investigated the epigenetic mechanisms associated with human sarcoma pathogenesis^5–8^. In the current study, we employed a model of human sarcoma generated *via* the spontaneous transformation of immortalized human bone marrow stromal cells (hBMSC) upon continuous passaging in culture. The hBMSC-Tum cells exhibited higher proliferation rates *in vitro* compared to parental control cells (Fig. 1a, upper left panel) and formed sarcoma-like tumors *in vivo* that were associated with high mitotic activity and increased angiogenesis (Fig. 1a, upper right panel). These tumors were positive for vimentin and negative for cytokeratin, confirming a mesenchymal origin (Fig. 1a, lower panels). Global gene expression profiling of the hBMSC-Tum cells revealed substantial changes in their transcriptome compared to the parental non-transformed hBMSC line (Fig. 1b). We identified 3269 genes that were differentially expressed (fold change ≥ 2.0; P (Corr), 0.05), which are shown in Supplementary Table 1. We found several of the upregulated genes in our study to be associated with different types of human sarcomas, including *GPR65*, *ARHGDIB*, *PPARG*, *HSD11B1*, *KYNU*, *SPRR2A*, *CCR1*, *PEG10*, *CLDN14*, *TFPI2*, *CYP2J2*, *PTPRN2*, *HLA-DQB1*, *CCL3*, *MAGEA8*, *COL5A3*, *HLA-DPA1*, *GFPT2*, *AIM2*, *HLA-DPB1*, and *TNNT1*^9,10^. Based on gene expression, the highest resemblance of hBMSC-Tum was with liposarcoma (7.5%), rhabdomyosarcoma (7.0%), malignant fibrous histiocytoma (6.3%), and fibrosarcoma (5.2%) (Supplementary Fig. 1). The distribution of the top-15 enriched pathways for the differentially-expressed genes in the hBMSC-Tum cells is shown in Fig. 1c and Supplementary Table 2. Several of the identified genetic pathways play important roles in stromal (mesenchymal) stem cell differentiation, such as ossification and adipogenesis, highlighting the maintenance of the stromal phenotype during transformation. We also observed the presence of an inflammatory signature in the hBMSC-Tum cells. The expression of a selected number of genes (*ACTB*, *LIN28B*, *HMGA2*, *IGF2BP2*, and *SLIT3*) was validated using qRT-PCR, demonstrating good concordance with the microarray data (Fig. 1d, upper panel). *LIN28B* was among the highly expressed genes based on the microarray data (approximately a 177.0 fold-change) and western blot analysis corroborated its upregulation in the hBMSCs-Tum cells (Fig. 1d, lower panel). We also noted the downregulation of CD24 and the upregulation of HLA-DR in the hBMSC-Tum cells, which was further validated by results from flow cytometry analysis (Fig. 1e). The expression of other hBMSC surface markers did not change significantly during transformation (Supplementary Fig. 2).

### Expression profiling of miRNA in hBMSC-Tum cells

**Figure 2:**
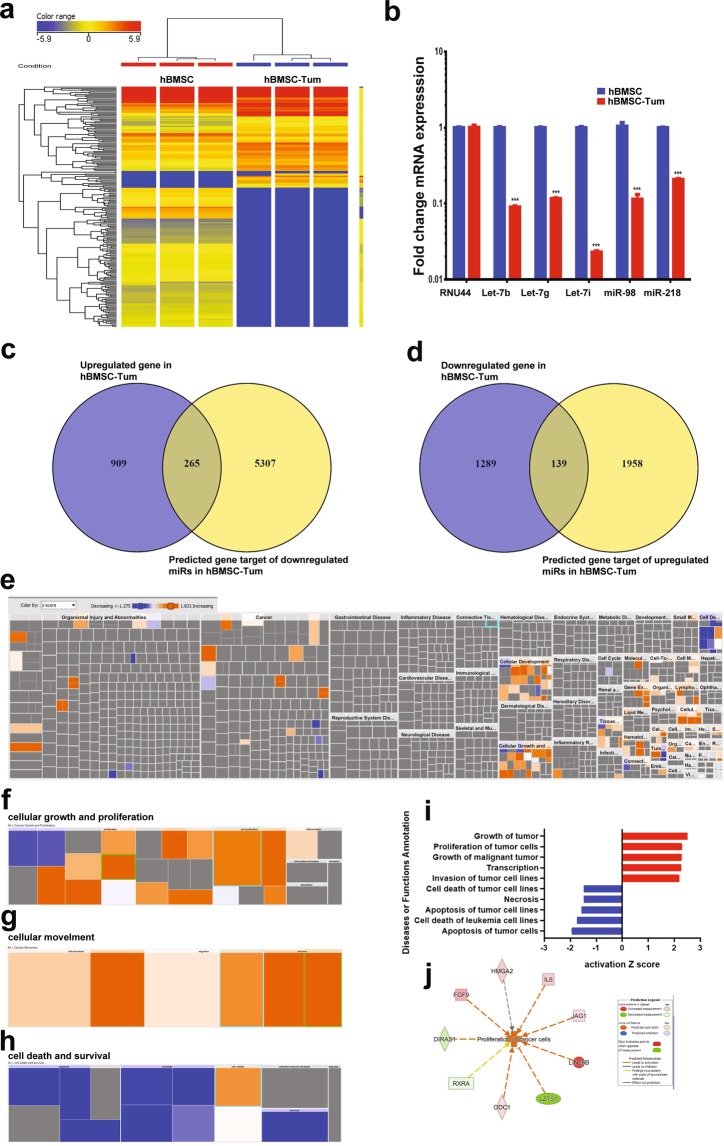
The expression profile of miRNA in the tumorigenic hBMSC-Tum cell line. (**a**) Hierarchical clustering of hBMSC-Tum cells compared to parental hBMSC cells based on miRNA expression levels. Each column represents a technical replica and each row represents an mRNA transcript. The expression level of each miRNA in a single sample is depicted according to the color scale. (**b**) Validation of the expression levels of miRNAs selected from the microarray data (RNU44, LET-7b, LET-7g, LET-7i, miR-98, and miR-218) using Taqman qRT-PCR. Data are presented as the mean ± S.E., n = 6 technical replicas. ***P < 0.0005. (**c**) Venn diagram depicting the overlap between the predicted gene targets for the downregulated miRNAs (based on TargetScan) versus the differentially upregulated genes in hBMSC-Tum cells compared to that in non-tumorigenic parental hBMSC cells. (**d**) Venn diagram depicting the overlap between the predicted gene targets for the upregulated miRNAs (based on TargetScan) versus the differentially downregulated genes in hBMSC-Tum cells compared to that in parental hBMSC cells. (**e**) Tree map (hierarchical heat map) depicting affected functional categories based on common genes from 2c and 2d where the major boxes represent a category of diseases and functions. (**f**) Cellular growth and proliferation, (**g**) cellular movements, and (**h**) represent cell death and survival functional category. Each individual colored rectangle is a particular biological function or disease and the color range indicates its predicted activation state: increasing (orange), or decreasing (blue). Darker colors indicate higher Z-scores. (**i**) Bargraph depicting top 5 activated and bottom 5 suppressed functional categories. X axis present the activation Z score. (**j**) Depicts the proliferation of cancer cells network.

To gain more insight into the epigenetic mechanisms leading to transformation of hBMSC-Tum cells, we performed global miRNA expression profiling of the hBMSC-Tum cells compared to non-transformed hBMSC cells. Cluster analysis revealed a distinct separation of the two cell lines based on their miRNA expression signatures (Fig. 2a). The list of differentially expressed miRNAs in the hBMSC-Tum cells compared to parental hBMSC cells is shown in Supplementary Table 3. The expression of a selected panel of miRNAs (Let-7b, Let-7g, Let-7i, miR-98, and miR-218) was subsequently validated using a Taqman microRNA assay and demonstrated good concordance with the microarray data (Fig. 2b). *In silico* prediction revealed approximately 22% of the upregulated genes to be potential targets of the downregulated miRNAs in the hBMSC-Tum cells (Fig. 2c). Similarly, approximately 10% of the downregulated genes were found to be potential targets of the upregulated miRNAs in the hBMSC-Tum cells (Fig. 2d). Common upregulated and downregulated genes from Fig. 2c,d were subsequently subjected to ingenuity pathway analysis (IPA), which provides a powerful tool to predict the increase or decrease in downstream biological activities and functions, which hare are likely to be casually affected by the transcriptome data. Figure 2e presents a high-level tree map of affected downstream functional categories based on common up and downregulated genes in hBMSC-Tum and predicted targets of differentially expressed miRNAs. This analysis revealed remarkable enrichment in several functional categories, mainly those involved in cancer cell growth, and proliferation and invasion (Fig. 2F,g). Additionally, genes associated with increased cell viability and survival were enriched, while genes associated with cell death were diminished (Fig. 2h). Top 5 enriched and top 5 diminished functional categories are shown in Fig. 2i. Growth and proliferation of cancer cells network is depicted in Fig. 2j, which highlighted a role for Lin28B and HMGA2 in this network. Upstream regulator analysis revealed significant enrichment in several mechanistic networks including SMARCA4, TNF, FOXO1, NFkB (complex), CAMP, Mek, CG, PPRC1, TGFB1, ERK, IL1B, PGR, and P38 MAPK (Supplementary Table 4).Taken together, our data revealed a significant increase in tumour growth, proliferation, and invasion, while functional categories associated with cell death were suppressed.

### LIN28B/LET-7 axis is associated with the transformed phenotype of hBMSC-Tum cells

**Figure 3:**
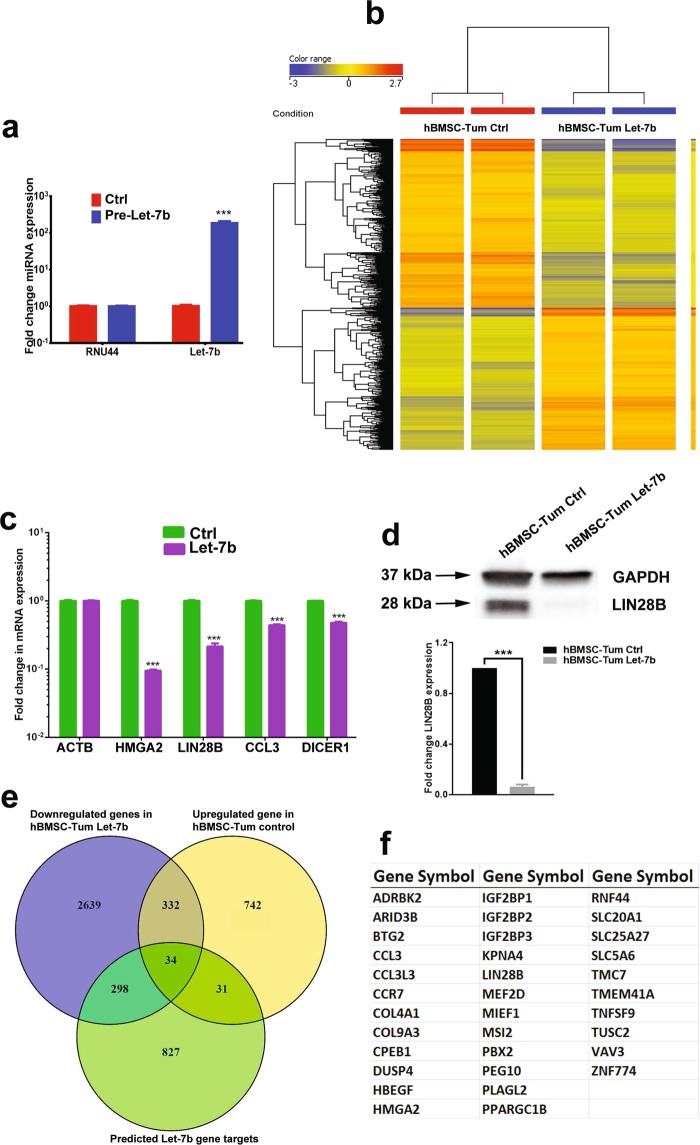
Changes in LET-7 expression regulates the transformed phenotype of the hBMSC-Tum cell line. (**a**) Measurement of the expression level by qRT-PCR of *LET-7b* in hBMSC-Tum cells transfected with pre-miR hsa-Let-7b. (**b**) Hierarchical clustering of hBMSC-Tum-Let-7b compared to that of hBMSC-Tum-control cells based on differentially expressed mRNA levels according to microarray analysis. Each column represents one technical replica and each row represents an mRNA transcript. The expression level of each gene in a single sample is depicted according to the color scale. (**c**) Validation of the expression levels of genes selected from the microarray data (*HMGA2*, *LIN28B*, *CCL3* and *DICER1*) using qRT-PCR. Data are presented as the mean ± S.E., n = 6 technical replicas. ***P < 0.0005. (**d**) Western blot analysis showing the downregulation of LIN28B protein expression in hBMSC-Tum-Let-7b cells compared to that in the control cells. Quantification of relative protein expression is shown form 3 technical replicas (**e**) Venn diagram depicting the overlap between the predicted gene targets for the *LET-7* family (based on TargetScan algorithm) compared to downregulated genes in hBMSC-Tum-LET-7b cells and the differentially upregulated genes in hBMSC-Tum cells. (**f**) List of 34 common genes from panel 3e.

Global mRNA and miRNA expression data revealed the upregulation of *LIN28B* and the downregulation of several members of the *LET-7* family in the hBMSC-Tum cells. LIN28B is known to inhibit processing and maturation of the LET-7 family miRNAs, while mature *LET-7* is also known to target LIN28B^11^. To investigate this plausible molecular mechanisms, we overexpressed *LET-7b* in hBMSC-Tum cells, which led to significant changes in their global gene-expression profile (Fig. 3a,b). Among the highly downregulated genes, *HMGA2*, *LIN28B*, *CCL3*, and *DICER1* were selected and validated using qRT-PCR (Fig. 3c). Downregulation of LIN28B in the hBMSC-Tum cells in response to *LET-7b* overexpression was also validated using western blot analysis (Fig. 3d). In order to identify the set of genes regulated by the LIN28B/LET-7 axis in in this system, we assessed the intersection between genes that were upregulated in the hBMSC-Tum cells and the genes downregulated in hBMSC-Tum cells transfected with *LET-7b*, and compared the results with the list of *in silico*-predicted LET-7b-target genes (Fig. 3e). This analysis revealed 34 genes that were common to all three conditions (Fig. 3e). *HMGA2*, *LIN28B*, and several members of the insulin-like growth factor-binding protein family were among the genes identified (Fig. 3f).

### The *LET-7* family directly targeted LIN28B and HMGA2 in hBMSC-Tum cells

**Figure 4:**
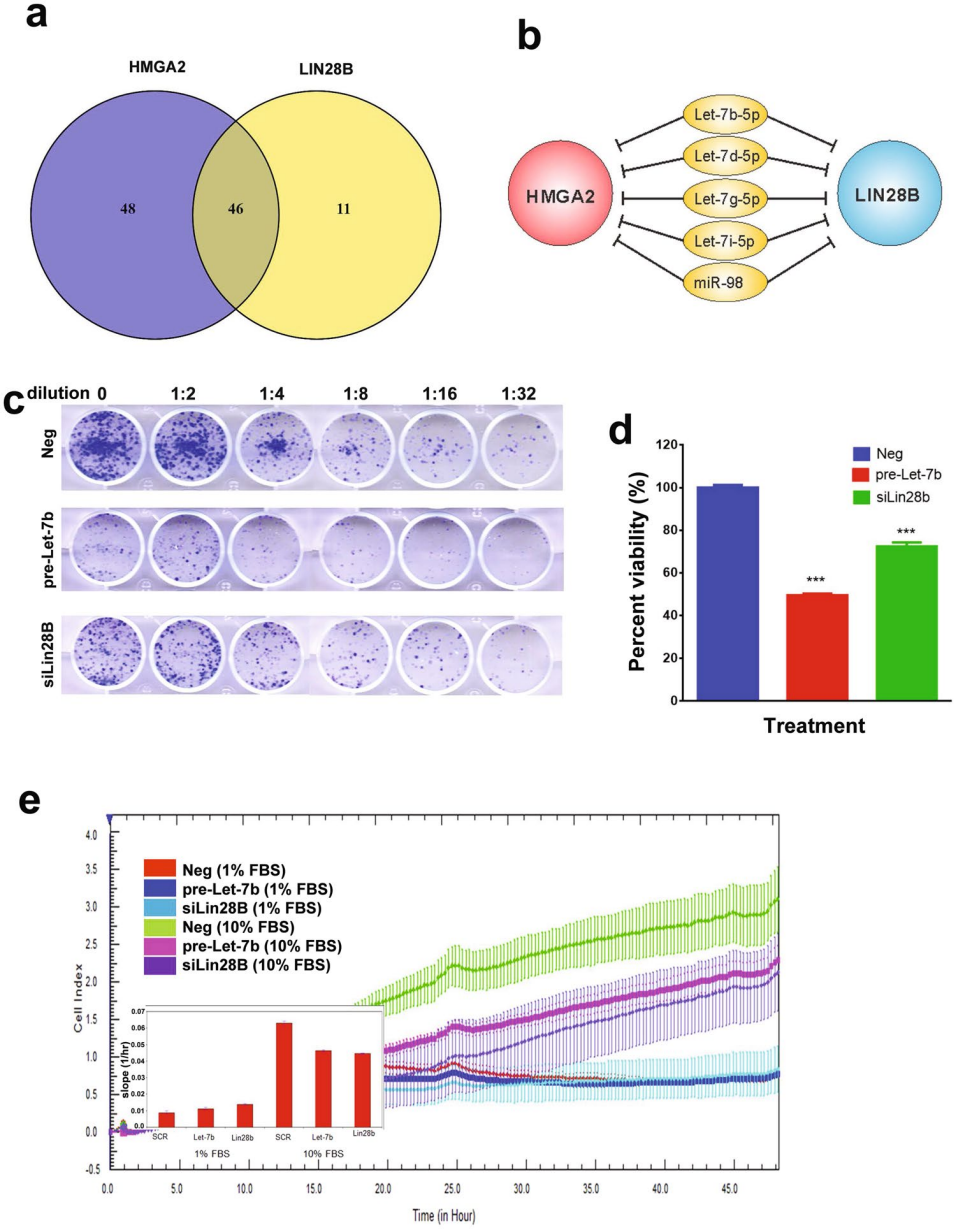
Changes in the LIN28B/LET-7 axis regulated the transformed phenotype of the hBMSC-Tum cell line. (**a**) Venn diagram depicting the overlap between miRNAs targeting *LIN28B* and *HMGA2* (*in silico*), which were downregulated in the hBMSC-Tum cells. (**b**) Illustration depicting the regulation of LIN28B and HMGA2 by the *LET-7* family, which are downregulated in the hBMSC-Tum cell line according to the current literature. Over expression of *LET-7b* or knockdown of *LIN28B* in the hBMSC-Tum cell line reduced the colony formation potential (**c**). For CFU assay, the initial seeding density was 1.5 × 10^4^ cells in the first well, followed by serial 2-fold dilution. (**d**) Cell viability was reduced as well. Data are presented as the mean ± S.E., n = 6, ***P < 0.0005. (**e**) Real time migration of different treatment groups toward 1% and 10% FBS-containing media using the RTCA system. Data are represented as mean ± S.D. from 3 technical replicas.

We subsequently focused on LIN28B and HMGA2 for further investigation given their documented roles in several human cancers^12^. Forty-six miRNAs downregulated in the hBMSC-Tum cells were also predicted by the *in silico* analysis to target *LIN28B* and *HMGA2* (Fig. 4a). Five of those miRNAs belonged to the *LET-7* family (Fig. 4b). Over expression of *LET-7b* (as a representative of the *LET-7* family), as well as siRNA-mediated knockdown of *LIN28B*, reduced colony formation (Fig. 4c) and proliferation (Fig. 4d) of the hBMSC-Tum cells. In addition, over-expression of LET-7b or siRNA-mediated knockdown of *LIN28B* expression led to significant inhibition of real-time migration of the hBMSC-Tum cells (Fig. 4e).

### Forced expression of LIN28B led to transformation of the parental hBMSC cells

**Figure 5:**
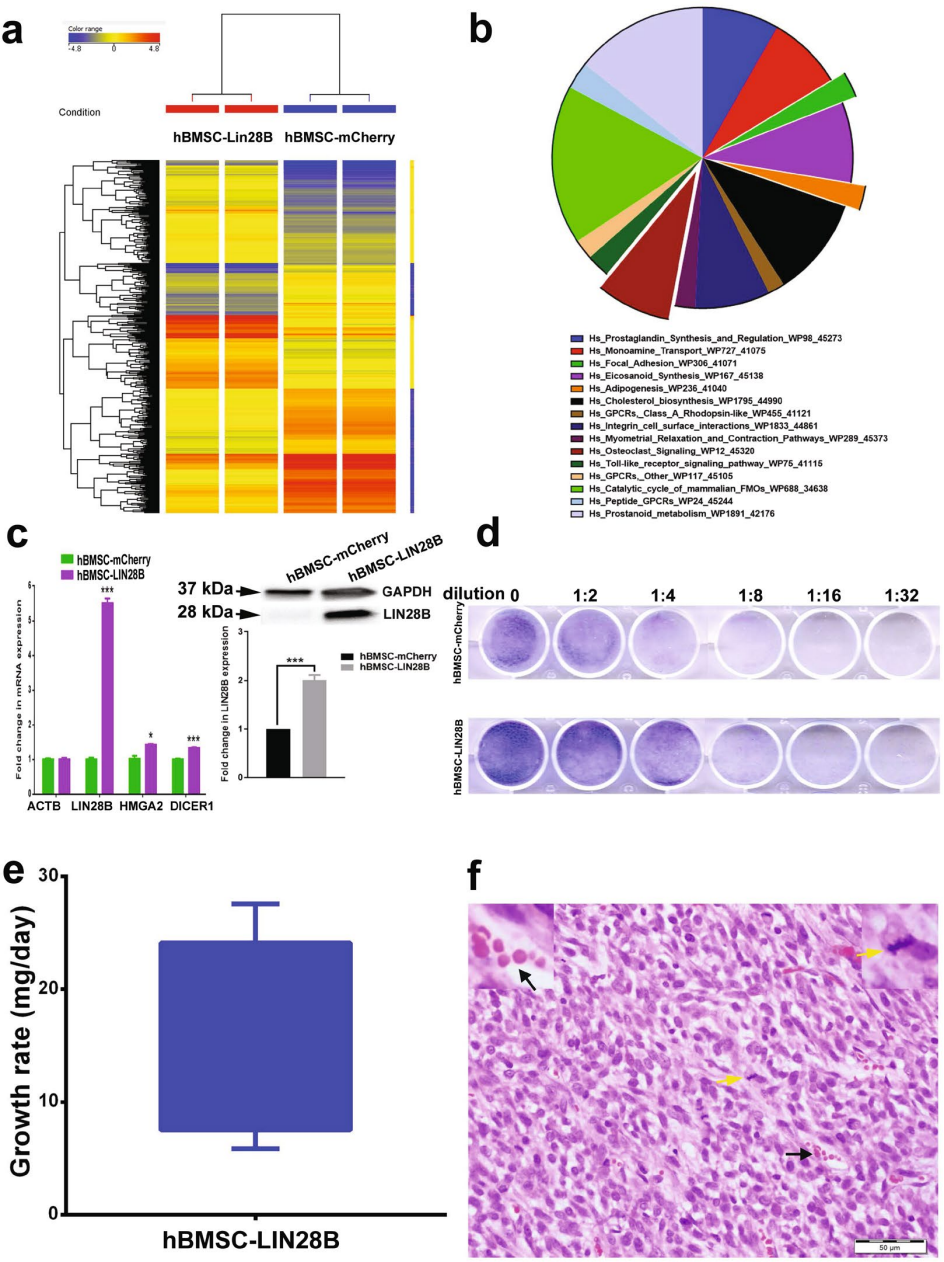
The parental hBMSC cell line overexpressing LIN28B (hBMSC-LIN28B) exhibited a transformed phenotype. (**a**) Hierarchical clustering of hBMSC-LIN28B compared to the control hBMSC-mCherry cells based on differentially expressed mRNA levels determined by microarray analysis. Each column represents one replica and each row represents an mRNA transcript. Expression level of each gene in a single sample is depicted according to the color scale. (**b**) Pie chart illustrating the distribution of the top-15 genetic pathways enriched in the hBMSC-LIN28B cells compared to that in the control hBMSC cells based on differentially expressed genes. The size of the chart wedge corresponds to fold-enrichment. (**c**) Expression levels of genes selected from the microarray data that were validated using qRT-PCR and western blot analysis. Quantification of relative protein expression is shown form 3 technical replicas. The hBMSC-LIN28B cell line exhibited enhanced *in vitro* colony formation (**d**). For CFU assay, the initial seeding density was 1.5 × 10^4^ cells in the first well, followed by serial 2-fold dilution. (**e**) The hBMSC-LIN28B cell line demonstrated tumor development in immunocompromised mice. Mice were implanted subcutaneously with hBMSC-LIN28B cells (mixed with matrigel) and on day 33, tumors were excised and weights were measured. Data on the y-axis are presented as tumor growth rate (mg/day) using the formula (tumor growth rate = weight (mg)/no. of days). (**f**) Histological analysis revealed sarcoma-like phenotype with the presence of many mitotic figures (yellow arrow) and angiogenesis (black arrow). Hematoxylin and eosin staining, 40×.

Our data highlighted a role for the LIN28B/LET-7 circuit in hBMSC-Tum cells. Therefore, we sought to determine if LIN28B could transform the parental hBMSC cells. Parental non-tumorigenic hBMSC line were transduced using lentiviral vector carrying human *LIN28B* and the global mRNA gene expression in the transduced cells was determined using microarray analysis. Hierarchical clustering comparing the hBMSC-LIN28B cells to the hBMSC-mCherry-control cells revealed a clear separation of the two cell lines (Fig. 5a). The top-15 enriched genetic pathways, based on the upregulated genes in the hBMSC-LIN28B cells, is shown in Fig. 5b and included those involved in focal adhesion, integrin cell surface interaction, G protein coupled receptors, and others. The expression levels of a number of these genes related to the LIN28B/LET-7 axis (LIN28B, HMGA2 and DICER1) were subsequently selected and validated using qRT-PCR (Fig. 5c). Expression of the LIN28B protein in the hBMSC-LIN28B cells was validated by western blot analysis, as shown in Fig. 5c. Forced expression of LIN28B enhanced the clonogenic potential of hBMSC cells (Fig. 5d). Furthermore, for *in vivo* assessment of tumorigenic potential, hBMSC-LIN28B cells were subcutaneously implanted into immune deficient SCID mice and tumor formation was monitored. Data presented in Fig. 5e demonstrates the progressive tumor growth of the implanted hBMSC-LIN28B cells, whereas the control cells did not form tumors. Histological examination of the tumors revealed proliferating fibromyxoid sarcoma with increased mitotic figures and angiogenesis (Fig. 5f).

### Differential sensitivity of hBMSC-Tum, hBMSC-LIN28B, and parental hBMSC cell lines to doxorubicin treatment

**Figure 6:**
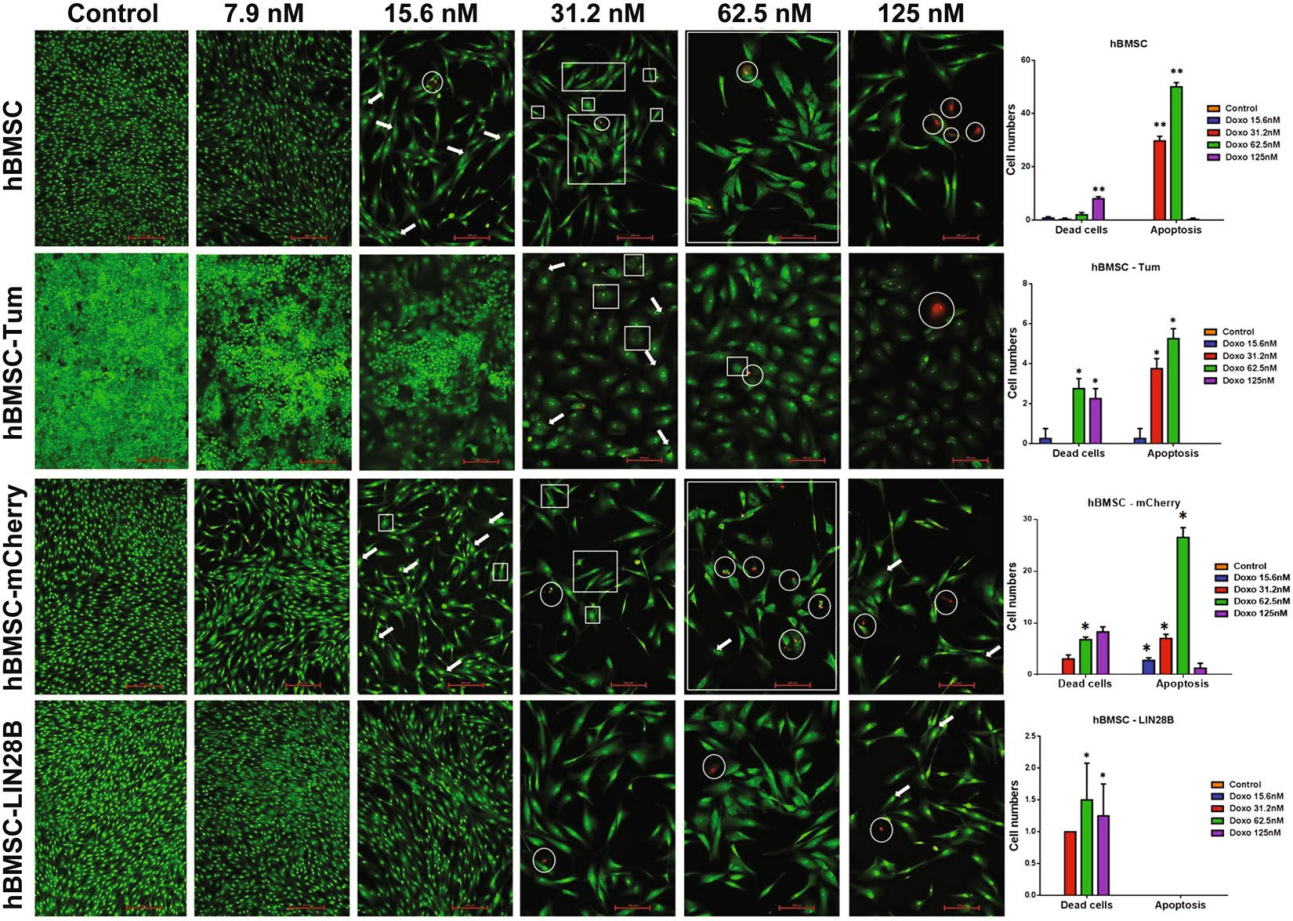
Differential sensitivity of cell lines hBMSC-Tum, hBMSC-LIN28B, and control hBMSC to doxorubicin treatment. Drug sensitivity of hBMSC-Tum and hBMSC-LIN28B cells and their respective control cells hBMSC and hBMSC-mCherry was assessed by doxorubicin treatment at concentrations ranging from 7.9 nM to 125 nM. Cells were stained with acridine orange and ethidium bromide in order to detect apoptotic cells (green) with condensed chromatin and apoptotic bodies, and dead cells (red). The arrow indicates the perinuclear cytoplasmic vacuoles, the square indicates apoptotic bodies, and the circle indicates dead cells (Scale bar = ***200 µm***). Data are representative of two replicas for each experimental condition, where more than 6 images were captured per condition. Quantification data from four representative images is presented as the mean ± S.E., n = 4. ***P < 0.0005.

Doxorubicin is an important chemotherapeutic agent in the treatment of sarcoma but a significant number of aggressive sarcomas exhibit doxorubicin resistance^13^. A number of studies has implicated the let-7 family in mediating chemotherapy resistance in other cancer types^14,15^. Therefore, we tested a possible role for LIN28B in doxorubicin resistance in sarcoma. We compared the chemo-sensitivity of hBMSC-LIN28B cells to doxorubicin to the sensitivity of hBMSC-Tum cells. Both types of cells were incubated in the presence of doxorubicin (7.8 nM to 125 nM) for 5 days and the apoptotic/necrotic responses were assessed. Doxorubicin concentrations >31.2 nM were toxic to all cells (Fig. 6). At 15.6 nM, hBMSC control cells exhibited formation of perinuclear cytoplasmic vacuoles. Compared to doxorubicin treatment at 31.2 nM, the number of cells containing apoptotic bodies (condensed chromatin and nuclear fragmentations) and dead cells was increased by treatment of the cells with 62.5 nM and 125 nM doxorubicin. However, these morphological changes were only observed in the hBMSC-Tum cells at the higher concentrations (Fig. 6, second panel). The hBMSC-LIN28B cells exhibited greater doxorubicin resistance compared to all the other cells types (Fig. 6, fourth panel). Similar differential sensitivity was also observed using flow cytometry cell cycle analysis (Supplementary Fig. 3).

### LIN28B expression was a possible prognostic marker for sarcoma

**Figure 7:**
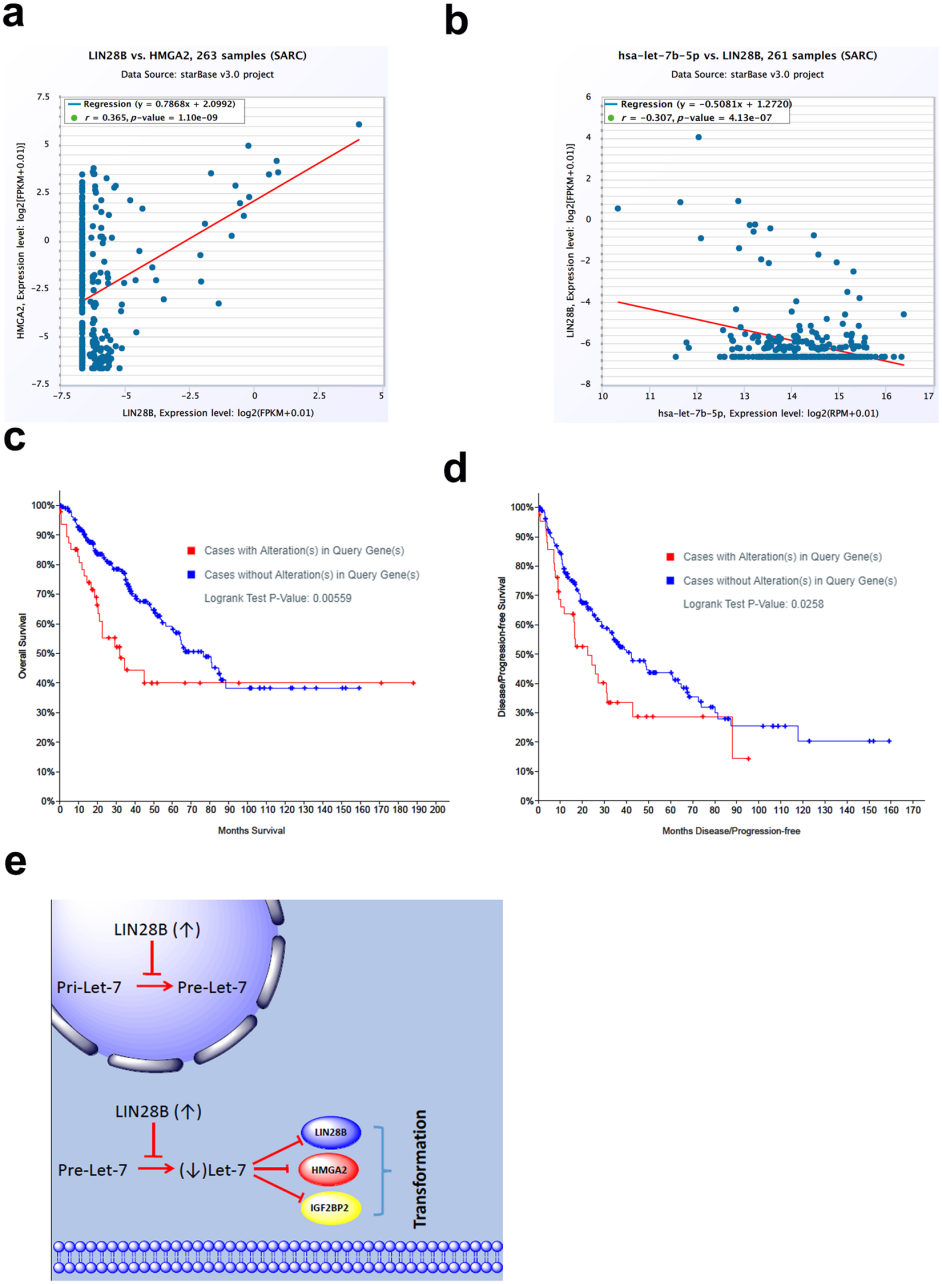
Expression of LIN28B and HMGA2 in sarcoma tissues were associated with a poor prognosis in patients. (**a**) Correlation between Lin28B and HMGA2 expression in a cohort of 263 sarcoma patients based on the starbase database. (**b**) Correlation between let-7b and Lin28B expression in 261 sarcoma patients based on the starbase database. Kaplan-Meier curves illustrate the duration of overall survival (OS) of 260 patients (**c**) and disease-free survival (DFS) of 231 patients (**d**) according to the expression of *LIN28B* and *HMGA2* in cohorts of patients in The Cancer Genome Atlas (TCGA) sarcoma dataset. Based on the log-rank analysis, expression of *LIN28B* and *HMGA2* were associated with poor OS (P = 0.005) and poor DFS (P = 0.02). (**e**) A proposed model for the role of the LIN28B/LET-7 axis in hBMSC transformation into sarcoma.

To corroborate the clinical relevance of our findings, we examined the expression of LIN28B and HMGA2 in sarcoma cohort using the StarBase database. Data presented in Fig. 7a revealed strong correlation between Lin28B and HMGA2 expression in 263 sarcoma patients (r = 0.36, p = 1.1 × 10^−9^). Additionally, inverse correlation between lET-7b and LIN28B was observed in the same cohort (r = −0.3, p = 4.1 × 10^−7^, Fig. 7b). Overall survival (OS, n = 260) and disease free survive l (DFS, n = 231) analysis on the TCGA sarcoma dataset revealed strong associations between the elevated expression levels of these proteins and poor OS (p = 0.005, Fig. 7c) and poor DFS (p = 0.02, Fig. 7d), thus implicating LIN28B and HMGA2 as clinically relevant biomarkers for sarcoma.

## Discussion

The notion that hMSCs being representative of the cell-of-origin of sarcomas has been considered in a number of recent studies; however, the molecular mechanisms leading to malignant transformation are still unknown. In the current study, we identified miRNA and mRNA expression signatures that were associated with the hBMSC transformed phenotype. In addition, we identified LIN28B/LET-7 as a possible molecular circuit underlying the transformed phenotype of hBMSC *in vitro* and associated it with the poor clinical prognosis for patients with sarcoma.

A number of previous models have been employed *in vitro* to study sarcoma biology, including transformed hMSCs using specific genetic elements^4^. In contrast to these studies, our study employed a biologically relevant model of spontaneously transformed hBMSCs and were able to compare non-transformed and transformed cells with the same genetic background. In addition, in contrast to the other models, the hBMSC-Tum model employed in current study maintained a normal karyotype^16^, helping to increase the sensitivity in identifying relevant molecular pathways associated with transformation. Finally, the relevance of this model was demonstrated by it exhibiting a similar molecular signature to those reported for clinically retrieved sarcomas^9^. Furthermore, our data revealed substantial similarities between the miRNA expression profile of transformed hBMSCs and those reported for human sarcomas. Approximately 30% of the altered miRNAs in human sarcomas reported by Subramanian *et al*.^8^ were also altered in transformed hBMSC-Tum cells identified in our current study and included hsa-miR-214, hsa-miR-379, hsa-miR-376a, hsa-miR-335, hsa-miR-10a, hsa-miR-29b, hsa-miR-29c, hsa-miR-30d, hsa-miR-143, hsa-miR-145, hsa-miR-195, hsa-miR-10b, hsa-let-7b, hsa-let-7d, hsa-miR-26a, hsa-miR-26b, hsa-miR-16, hsa-miR-15a, hsa-miR-497, hsa-miR-222, hsa-miR-424, hsa-miR-21, hsa-miR-218, and hsa-miR-30c.

Cumulative evidence implicates miRNAs and their regulatory networks in arbitrating tumor progression, mainly through the regulation of their relevant mRNA targets^17^. LIN28A and LIN28B selectively inhibit LET-7 miRNA expression and biogenesis in embryonic stem cells (ESCs), and block the expression of *LET-7* through TUTase-dependent and independent mechanisms^18,19^. In addition, compelling evidence supports the role for *LIN28* as an oncogene, including its direct targeting by the *LET-7* family and its effect in combination with several known oncogenes on cell reprogramming^11,20,21^. In addition, LIN28A and LIN28B are upregulated in several advanced human cancers and are reliable predictors of a poor prognosis^12,21^. However, the role of the LIN28/LET-7 axis in hBMSC transformation and sarcoma pathogenesis remains unknown.

We observed the upregulation of LIN28B and downregulation of the *LET-7* family in the tumorigenic transformation of hBMSCs. The tumor-suppressive *LET-7* miRNA family targets oncogenes such as *HMGA2*, *LIN28B*, and *IGF2BP1* to form a *LET-7* antagonizing, self-promoting, oncogenic triangle^22^. Concordantly, our data demonstrated that HMGA2 and LIN28B were upregulated in hBMSC-Tum cells and were suppressed upon the re-expression of *LET-7*. Iliopoulos *et al*. reported that the activation of NFKB by Src induces cellular transformation *via* the LIN28B, LET-7, and IL-6 circuit^23^. The hBMSC-Tum model exhibited high levels of pro-inflammatory markers such as IL1β and IL6. Therefore, it is plausible that high levels of inflammation during the transformation of parental hBMSC cells induces the expression of LIN28B, followed by the downregulation of *LET-7* and increased expression levels of IL6, which ultimately results in a transformed phenotype. Thus, we propose a model for the role for the LIN28B/LET-7 axis in the underlying mechanism of hBMSC transformation (Fig. 7e). Concordant with our findings, interrogation of LIN28B expression in the Cancer Cell Line Encyclopedia (CCLE) database revealed upregulated expression of LIN28B in a number of Ewing’s sarcoma cell lines (Supplementary Figure 4). While our proposed model highlights a role for LIN28B in cellular transformation *via LET-7*, it is plausible that LIN28B may also promote cellular transformation through a *LET-7* independent mechanism^24^.

Our data highlighted a role for LIN28B in conferring doxorubicin resistance *in vitro*. Edmonson *et al*. reported a lower efficacy of doxorubicin when it is administered alone compared to when it is combined with ifosfamide or with mitomycin and cisplatin, suggesting the presence of a resistance mechanism in patients with advanced soft tissue sarcomas^13^. Therefore, it is possible that elevated levels of LIN28B indeed mediate doxorubicin resistance in those patients. In agreement with those findings, patient survival data positively correlated elevated levels of LIN28B and HMGA2 with poor clinical outcome in sarcoma patients. Our data support the clinical relevance of LIN28B and HMGA2 as possible prognostic markers and as molecular targets for novel therapeutic approaches in sarcoma.

## Materials and Methods

### Ethics statement

All animal procedures were approved by the local ethical committees on animal experiments at the University of Southern Denmark. All methods were performed in accordance with the relevant guidelines and regulations of the animal care committee at the University of Southern Denmark.

### Cell lines and culture

We used a well-characterized hTERT-immortalized adult hBMSC cell line that expressed all the known markers of primary hBMSCs and is able to form bone and establish the bone marrow microenvironment following *in vivo* implantation at early population doublings^2,25,26^. For simplicity, will refer to this model as hBMSC for the remained of this manuscript. We also used its transformed counterpart (hBMSC-Tum), which spontaneously arose during long-term culturing^27^. Both cell lines were cultured in Dulbecco’s modified Eagle’s medium (DMEM) supplemented with 4500 mg/l d-glucose, 4 mM l-glutamine, 110 mg/l sodium pyruvate, 10% fetal bovine serum (FBS), 1% penicillin–streptomycin, and nonessential amino acids as previously described^2^.

### Lentiviral transduction and pre-miR transfection

Transduction was performed in accordance with our previously published protocols^28^. Briefly, lentiviral particles encoding for human LIN28B (LP-OL05603-LX304-0200-S) or control lentiviral particles mCherry (LP-MCHR-LV105-0205) were purchased from Genecopoeia Inc. (Rockville, MD, USA). The hBMSC cells (1.5 × 10^5^) were seeded into 24-well plates with complete DMEM. Twenty-four hours later, after the cells had obtained approximately 80% confluency, the medium was removed and 20 μl of crude lentiviral particles in 500 μl of DMEM + 5% heat-inactivated serum (Invitrogen) and 1% Pen–Strep supplemented with polybrene (8 μg/ml; Sigma, St. Louis, MO, USA) were added to the cells. Seventy-two hours later, the medium was removed and transduced cells were selected with puromycin (1 μg/ml; Sigma, St. Louis, MO, USA) for 1 week or until stably transduced cells were generated.

The pre-miR-negative control, pre-miR hsa-let-7b-5p, scrambled siRNA control, and siRNA targeting human LIN28B were purchased from Applied Biosystems (Invitrogen, Carlsbad, CA, USA). Transfection was performed using a reverse transfection approach as previously described^17^. Briefly, pre-miRs or siRNA at a final concentration of 30 nM was diluted in 50 μl of Opti-MEM (11058-021; Gibco, Carlsbad, CA, USA), and 1 μl of Lipofectamine 2000 (catalogue No. 52758; Invitrogen) was diluted in 50 μl OPTI-MEM. The diluted pre-miR, siRNA, and Lipofectamine 2000 were mixed and incubated at ambient temperature for 20 min. Twenty microliters of transfection mixture was added to the tissue culture plate and subsequently 10,000 cells in 60 μl transfection medium (complete DMEM without antibiotics) were added to each well. Twenty-four hours later, the transfection cocktail was replaced with complete DMEM.

### Gene and miRNA expression profiling

RNA isolation, and the expression profiling of genes and miRNA were performed according to our previously published protocols^17,28,29^. In brief, total RNA was isolated using a Total Tissue RNA Purification Kit from Norgen-Biotek Corp. (Thorold, ON, Canada) and was quantified using a NanoDrop 2000 spectrophotometer (Thermo Scientific, Wilmington, DE, USA). Total RNA was labeled and then hybridized with an Agilent Human SurePrint G3 Human GE 8 × 60 k or Human 8 × 60 k miRNA microarray chip (Agilent Technologies, Palo Alto, CA, USA). All microarray experiments were conducted at the Microarray Core Facility in the Stem Cell Unit, Department of Anatomy, King Saud University College of Medicine. Data were subsequently normalized and analyzed using GeneSpring 13.0 software (Agilent Technologies). Pathway analyses were conducted using the Single Experiment Pathway analysis feature in GeneSpring 13.0. A twofold cut-off with P < 0.05 was used. Target prediction was conducted using the TargetScan database and the built-in feature of GeneSpring 13.0.

### Gene set enrichment and modeling of gene interactions networks

Up and downregulated genes which are predicted targets of differentially expressed miRNAs in the hBMSC-Tum line were imported into the Ingenuity Pathways Analysis (IPA) software (Ingenuity Systems; www.ingenuity.com/) and were subjected to functional annotations and regulatory network analysis using upstream regulator analysis (URA), downstream effects analysis (DEA), mechanistic networks (MN) and causal network analysis (CNA) prediction algorithms. The p value is the negative log of P and represents the possibility that focus genes in the network being found together by chance. Z score corresponds to the enrichment of particular functional category^30,31^.

### mRNA and miRNA validation by qRT-PCR

Expression levels of the mRNAs were validated using SYBR Green-based quantitative reverse transcriptase-polymerase chain reaction (qRT-PCR) with an Applied Biosystems ViiA™ 7 Real-Time PCR System as previously described^17^. The total RNA (500 ng) was reverse transcribed into complementary DNA (cDNA) using a High Capacity cDNA Reverse Transcript Kit (catalogue No. 4368814; ABI) according to the manufacturer’s protocol. Relative levels of mRNA were determined using the cDNA as template in real-time PCR analysis using the Applied Biosystems ViiA 7 System. Primer sequences used in the current study are listed in Table 1. The relative expression levels were calculated using the −ΔΔCT method. The housekeeping gene for β-actin was used as an endogenous control. For miRNA validation, 10 ng of total RNA was reverse transcribed using a TaqMan MicroRNA Reverse Transcription Kit (catalogue No. 4366596; ABI) and the relative miRNA expression levels were determined using TaqMan Universal Master Mix II, no UNG (catalogue No. 4440040; ABI). The relative expression levels were calculated using the −ΔΔCT method. RNU44 was used as an endogenous control.

**Table 1:** List of SYBR green primers used in current study.

No	Name	Sequence
1	ACTB	F 5′TCAAGATCATTGCTCCTCCTGAG
		R 5′ACATCTGCTGGAAGGTGGACA
2	LIN28B	F 5′ GGCCTTGAGTCAATACGGGT
		R 5′ GGCACTTCTTTGGCTGAGGA
3	HMGA2	F 5′CAGACCTAGGAAATGGCCACAACAA
		R 5′AAATCGAACGTTGGCGCCCC
4	IGF2BP2	F 5′CTGGCCGTGTTCCGGGAGAA
		R 5′TTCCTGTTGGCAGGGAGTCCTGG
5	SLIT3	F 5′CCGCCTAACTACACAGGTGAGCTAT
		R 5′CGCTGTAGCCAGGGACACACT
6	CCL3	F 5′AAGGACACGGGCAGCAGACA
		R 5′AGCAGCAAGTGATGCAGAGAACTGG
7	DICER1	F 5′ATGACCCCTGCTTCCTCAC
		R 5′TCTTCCCTGAGCCAGTGTTT

### Immunophenotyping by flow cytometry (FACS)

Immunophenotypic analysis was performed in accordance with our previously published protocols^32^. In brief, cells were harvested and washed twice in ice-cold phosphate buffered saline (PBS) supplemented with 0.5% bovine serum albumin and resuspended at 10^6^ cells/ml. Then, 10 μl of mouse anti-human CD24-FITC and HLADR-PE conjugated antibodies (from BD Biosciences, San Jose, CA, USA) was added to 100 μl of cell suspension. Cells were incubated for 30 min at 4 °C in the dark, washed with PBS, resuspended in 500 μl of PBS, and analyzed using a Navios flow cytometer (Beckman Coulter, Brea, CA, USA). Living cells were gated in a dot plot of forward vs. side scatter signals obtained on linear scale after being analyzed with appropriate negative control using FITC and PE-conjugated mouse IgG1 isotype control for nonspecific background. Minimum 5000-gated events were acquired on a log fluorescence scale. Data were further analyzed using Kaluza software (1.2 version, Beckman Coulter). For cell cycle analysis, cell pellets were collected and washed with phosphate-buffered saline (PBS), and then were resuspended in 1 ml of FACS buffer (PBS/0.5% BSA), and then 3 ml of ice-cold 70% ethanol was added to fix the cells for 1 h on ice. Cell pellets were subsequently centrifuged, and re-suspended in 500 μL of PBS supplemented with 40 µg/mL RNAse A (Sigma) and 50 µg/mL propidium iodine (PI), before being analyzed using a Navious flow cytometer (Beckman Coulter, Miami, FL, USA). Staining was detected in the green fluorescence channel (FL1) and the data were analyzed by Kaluza software (Beckman Coulter).

### Cell migration

Real-time measurement of cell migration was performed using the xCELLigence RTCA DP system (ACEA Biosciences, San Diego, CA) as described previously^28^. Briefly, cells were starved for 24 h in media containing 1% serum, followed by seeding 8.0 × 10^4^ cells per well in 16-well microelectronic sensor plates with two-chamber trans-well inserts (CIM-plate insert; ACEA Bioscience) containing the appropriate serum conditions. Medium containing 10% serum (chemo-attractant) or 1% serum (control) was added to the bottom chambers of the wells.

### Measurement of cell viability and colony formation

Viability of the negative control cells compared to the pre-miR Let-7b or si-LIN28B cells (hBMSC-Tum) was determined using an alamarBlue assay as previously described^28^. All assays were carried out with the appropriate controls. Briefly, 1 × 10^4^ cells were cultured in 96-well plates and the cell viability measured at the indicated time points by adding 10% volume alamarBlue assay reagent and measuring the fluorescence at excitation and emission wavelengths of 530 nm and 590 nm, respectively.

The colony forming ability of the control cells compared to the pre-miR Let-7b or si-LIN28B cells (hBMSC-Tum), hBMSC-LIN28B cells, and hBMSC-mCherry cells was determined using a clonogenic assay as previously described^29^. Briefly, cells were seeded into 12-well plates at different concentrations of serial diluted cells (1:2 to 1:64), with an initial seeding density of 1.5 × 10^4^ cells per well and incubated at 37 °C under 5% CO_2_ for 10 d. The plates were then washed and stained using a Diff-Quik staining set (Siemens Healthcare Diagnostics). The plates were scanned and the number of colonies was evaluated under light microscopy.

### Western blot analysis

Cells were lysed using RIPA buffer (Pierce Inc, Thermo Scientific, USA) containing 1 × Halt Protease Inhibitor Cocktail (Pierce Inc., Thermo Scientific). Samples containing 30 µg of total protein were electrophoretically separated and blotted using the Bio-Rad V3 Western Workflow system according to the manufacturer’s recommendation. Immunoblotting was performed using anti-LIN28B rabbit monoclonal antibody (Ab191881, 1:2000; Abcam, Cambridge, MA). The primary antibody was incubated overnight at 4 °C. Horseradish peroxidase (HRP)-conjugated goat anti-rabbit was used as the secondary antibody, whereas HRP-conjugated anti-GAPDH (glyceraldehyde-3-phosphate dehydrogenase) antibody (1:1000; Invitrogen) was used as a loading control. Chemiluminescent detection was performed using WesternSure Chemiluminescent Substrate (LI-COR, Lincoln, NE, USA). Band intensities were quantified using the band quantification tool in Image Laboratory 5.0 software (Bio-Rad Laboratories).

### Measurement of apoptosis and cell death

Fluorescence-based analysis of apoptosis and the evaluation of dead cells were performed after exposure of the cells to different concentrations of doxorubicin (7.8 nM to 125 nM), using an acridine orange and ethidium bromide (AO/EB) staining method as previously described^32^. Briefly, after treatment, the two groups of cells, hBMSC control *vs*. hBMSC-Tum and hBMSC-mCherry control *vs*. hBMSC-LIN28B cells were stained with dual fluorescent staining solution (1 µl) containing 100 µg/ml AO and 100 µg/ml EB (AO/EB, Sigma, St. Louis, MO). Cells were washed twice with PBS and gently mixed with AO/EB dye solution (1:100) for 1 min. After staining, the cells were evaluated and photographed using a Nikon Eclipse Ti fluorescence microscope and quantified. Cells cultured without drug were considered experimental controls. AO/EB staining uses the combination of two dyes to visualize cells with aberrant chromatin organization, apoptotic bodies, cytoplasmic changes, and dead cells. The differential uptake of AO/EB allows for the identification of viable and non-viable cells.

### *In vivo* tumorigenicity assays in immune deficient SCID mice

*In vivo* tumor formation was performed as previously described^27^. Briefly, six- to eight-week-old female immunodeficient mice (NOD.CB17-Prkdc^scid^/J) were utilized in the xenograft experiments. Ten million hBMSC-mCherry or hBMSC-LIN28B cells were mixed 1:1 with Matrigel (BD Biosciences, San Jose, CA, USA) and subcutaneously injected into the right flank of SCID mice. Tumor mass was assessed on day 33 post implantation. At the end of the experiment, tumors were excised, fixed in 10% buffered formalin, embedded in paraffin, and sectioned on a microtome. Sections were then stained with hematoxylin and eosin.

### Expression and survival analyses of The Cancer Genome Atlas sarcoma dataset

Correlation between the expression of LIN28B, HMGA2, and LET-7b in sarcoma patients was assessed using the starBase Pan-Cancer database^33^. Kaplan-Meier curve analysis for the expression of LIN28B and HMGA2 in The Cancer Genome Atlas (TCGA) sarcoma dataset, which included 260 patients for overall survival (OS) and 231 patients for disease-free survival (DFS), was conducted as previously described^32^. The log-rank test was used to determine statistical significance in the curve comparison analysis.

### Statistical analysis

Statistical analyses and graph generation were performed using Microsoft Excel 2010 and GraphPad Prism 6.0 software (GraphPad, San Diego, CA, USA). P-values were calculated using the two-tailed t test. P value < 0.05 was considered significant.

## Supplementary information

Supplementary figures

Supplementary Table 1

Supplementary Table 2

Supplementary Table 3

Supplementary Table 4

## Data Availability

Processed data are provided as supplementary datasets.
