# Supplementary material for: Neoplastic Transformation of Human Mesenchymal Stromal Cells Mediated via LIN28B
Source: Sci Rep. 2019 May 30;9:8101. doi: 10.1038/s41598-019-44536-1 (PMC6542832; doi:10.1038/s41598-019-44536-1)
Supplement: Supplementary file 1 — Supplementary figures [file 41598_2019_44536_MOESM1_ESM.pdf]

Neoplastic Transformation of Human Mesenchymal Stromal Cells Mediated via *LIN28B*

Radhakrishnan Vishnubalaji, Ramesh Elango, Mashael Al-Toub, Muthurangan Manikandan, Ammar Al-Rikabi, Linda Harkness, Nicholas Ditzel, Muhammad Atteya, Rimi Hamam, Musaad Alfayez, Abdullah Aldahmash, Moustapha Kassem, & Nehad M. Alajez

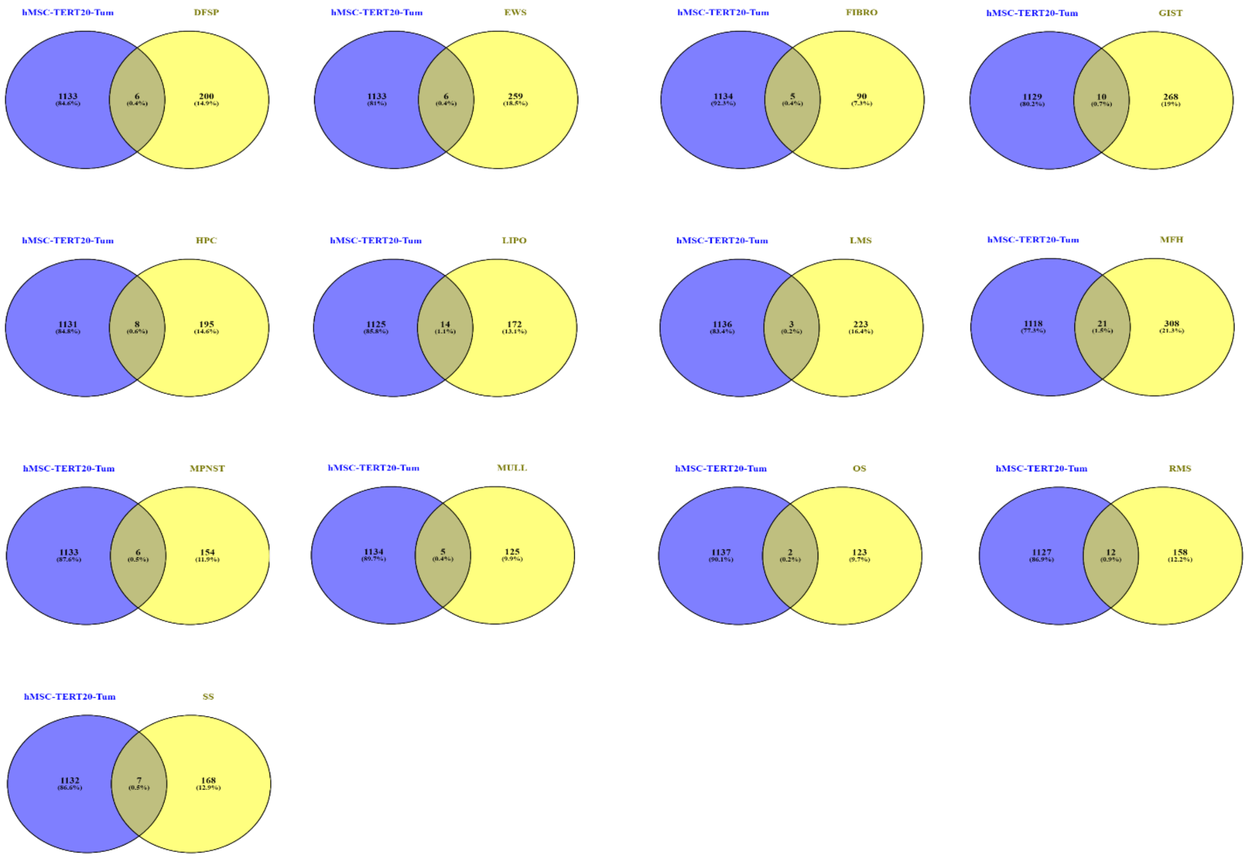

**Type**	**% resemblance**
**LIPO**	**7.5**
**RMS**	**7**
**MFH**	**6.3**
**FIBRO**	**5.2**
SS	4
MULL	3.8
MPNST	3.7
GIST	3.5
DFSP	2.9
HPC	2.7
EWS	2.2
OS	1.6
LMS	1.3

Supplementary figure1. Comparison of gene upregulated in ThMSC-TERT20-Tum compared to gene signatures associated with different human sarcomas. DFSP: dermatofibrosarcoma; EWS: Ewing's sarcoma; FIBRO: Fibrosarcoma; GIST: gastrointestinal stromal tumor; HPC: hemangiopericytoma; LIPO: Liposarcoma; LMS: leiomyosarcoma; MFH: Malignant fibrous histiocytoma; MPNST: malignant peripheral nerve sheath tumor; MULL: mixed mullerian; OS: osteosarcoma; RMS: Rhabdomyosarcoma; SS: synovial sarcoma.

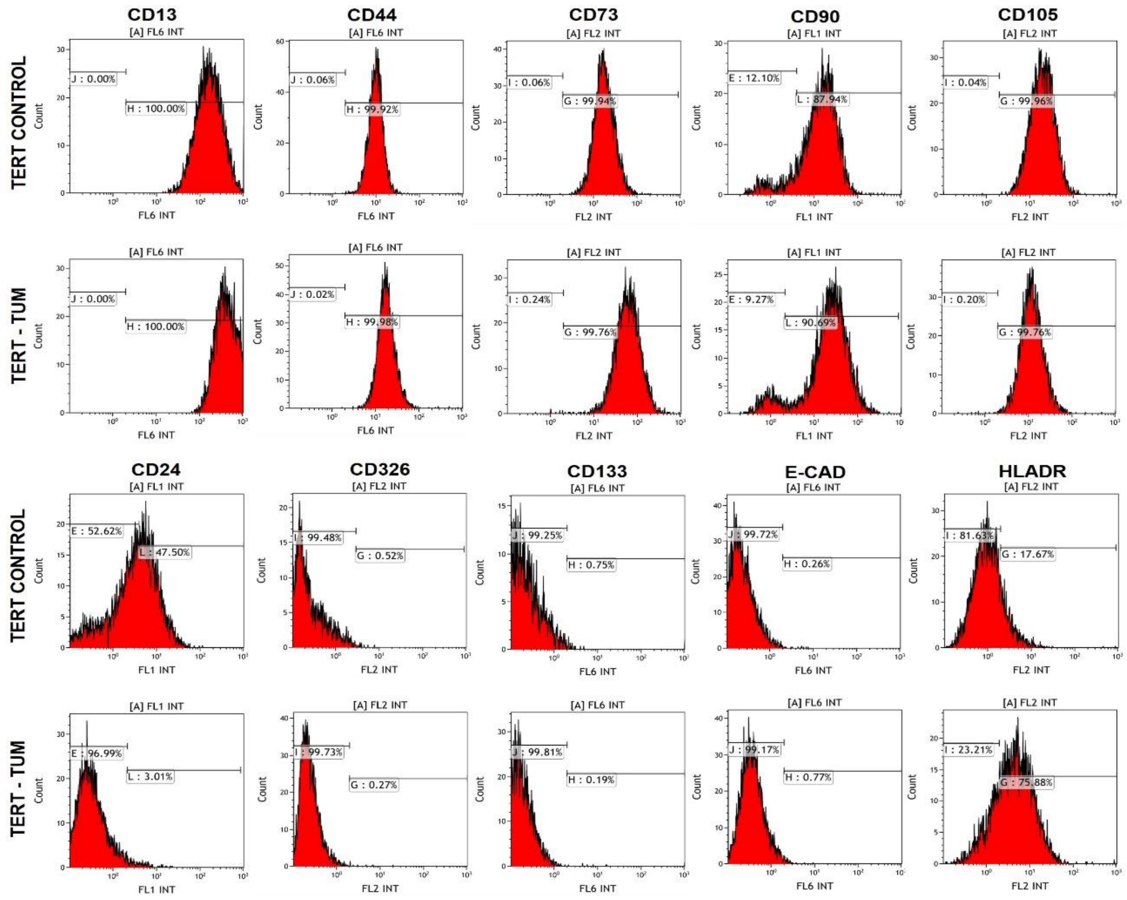

Supplementary figure2. The expression of hMSC surface markers on hMSC-TERT-Tum and parental hMSC-TERT cells. The expression of indicated surface markers were measured on the surface of hMSC-TERT and hMSC-TERT-Tum cells using flow cytometry. Data were analyzed using Kaluza analysis software and were plotted as histograms.

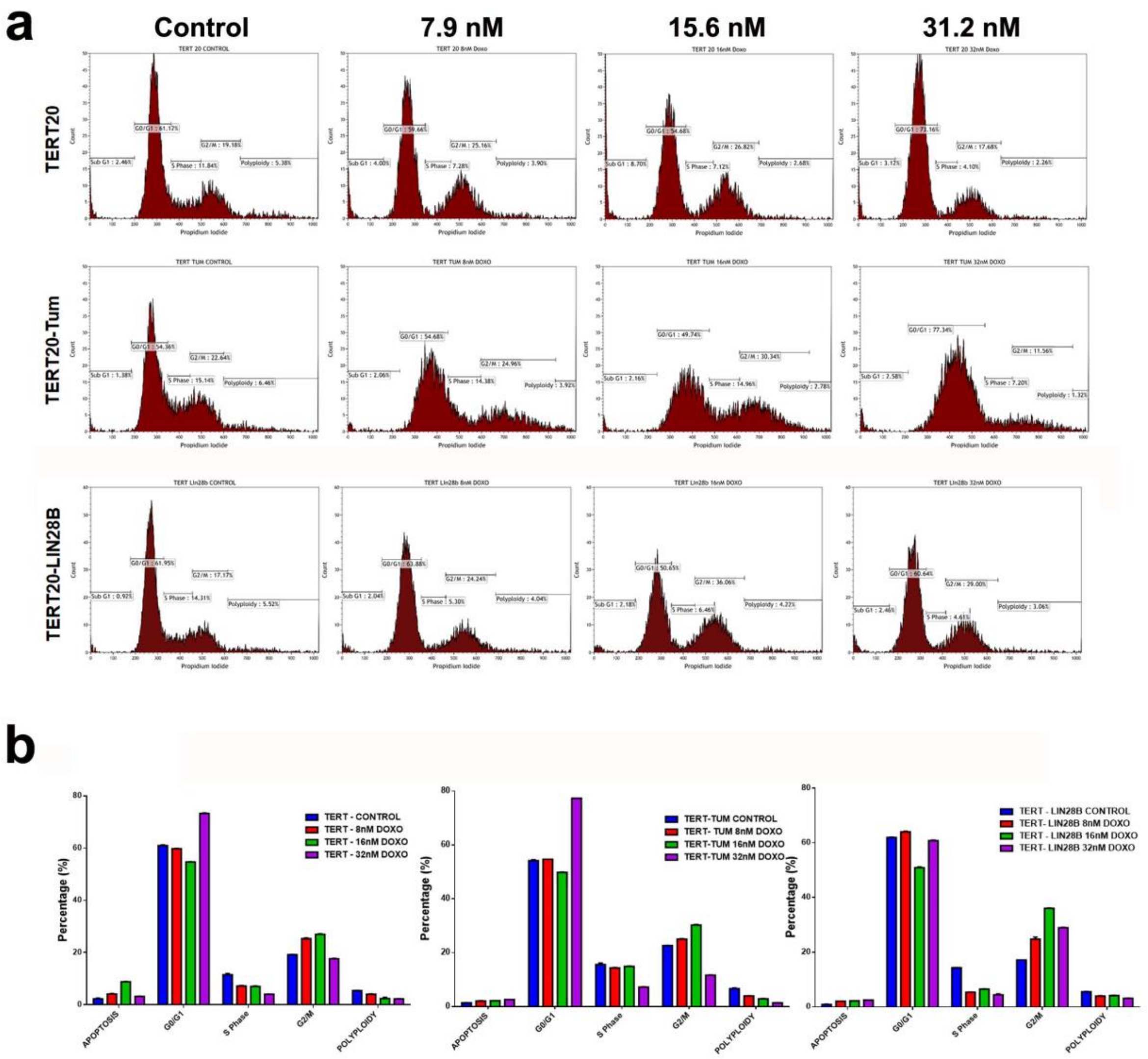

Supplementary figure 3. Cell cycle analysis of hMSC-TERT20, hMSC-TERT20-Tum, and hMSC-TERT20-Lin28B to Doxorubicin treatment. Cells were treated with different concentration of Doxorubicin (8, 16 and 32 nM) for three days. Cells were subsequently fixed in 70% ethanol and stained with 50 µg/mL propidium iodide, and then acquired using the Navious Flow Cytometer (Beckman Coulter, Miami, Florida, USA). Samples were analyzed using Kaluza software (Beckman Coulter). Data are shown as histogram (a) or as quantification from three replicates (b).

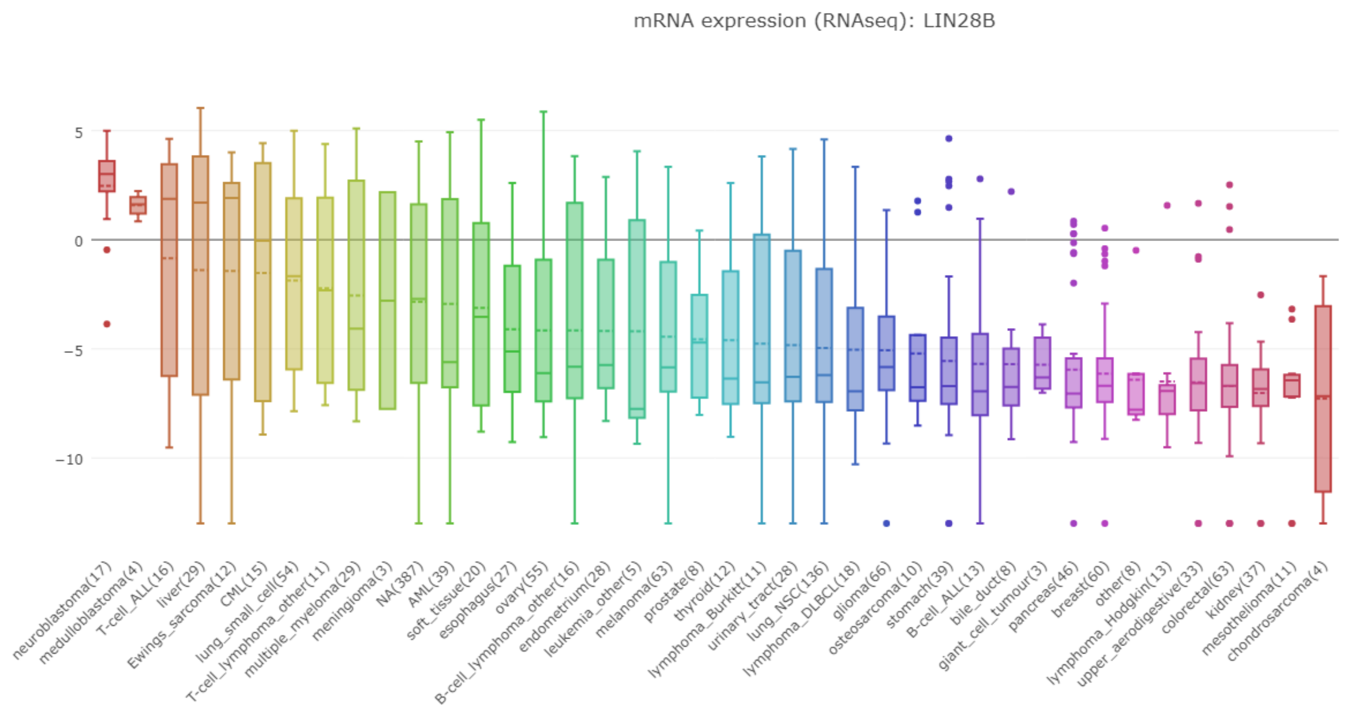

Supplementary figure 4. Expression of Lin28B in a panel of human cancer cell lines. The expression of Lin28B mRNA was retrieved from the Cancer Cell Line Encyclopedia (CCLE) RNAseq database. X-axis represent cancer cell type while y-axis indicates Lin28B mRNA expression level.
